# Supplementary material for: Unveiling the Bioactive Potential of the Invasive Jellyfish Phyllorhiza punctata Through Integrative Transcriptomic and Proteomic Analyses
Source: Biomolecules. 2025 Aug 4;15(8):1121. doi: 10.3390/biom15081121 (PMC12383608; doi:10.3390/biom15081121)
Supplement: Supplementary file 1 [file biomolecules-15-01121-s001.zip › Suplementary Material/FileS3/PEPFOLD4/XP_065057019_7528/clusters.html]

 PEP-FOLD clusters


Download archive of all models  here   

|  |  |  |
| --- | --- | --- |
| # | file | sOPEP |
| # cluster 1 - Size: 83 |  |  |
|  | XP\_065057019\_7528-model1 | -19.158 |
|  | XP\_065057019\_7528-model1.1 | -19.138 |
|  | XP\_065057019\_7528-model1.2 | -18.9463 |
|  | XP\_065057019\_7528-model1.3 | -18.8675 |
|  | XP\_065057019\_7528-model1.4 | -18.8664 |
|  | XP\_065057019\_7528-model1.5 | -18.8352 |
|  | XP\_065057019\_7528-model1.6 | -18.8246 |
|  | XP\_065057019\_7528-model1.7 | -18.7999 |
|  | XP\_065057019\_7528-model1.8 | -18.7875 |
|  | XP\_065057019\_7528-model1.9 | -18.7191 |
|  | XP\_065057019\_7528-model1.10 | -18.7129 |
|  | XP\_065057019\_7528-model1.11 | -18.701 |
|  | XP\_065057019\_7528-model1.12 | -18.6991 |
|  | XP\_065057019\_7528-model1.13 | -18.6991 |
|  | XP\_065057019\_7528-model1.14 | -18.6948 |
|  | XP\_065057019\_7528-model1.15 | -18.687 |
|  | XP\_065057019\_7528-model1.16 | -18.6709 |
|  | XP\_065057019\_7528-model1.17 | -18.6584 |
|  | XP\_065057019\_7528-model1.18 | -18.6584 |
|  | XP\_065057019\_7528-model1.19 | -18.6506 |
|  | XP\_065057019\_7528-model1.20 | -18.6506 |
|  | XP\_065057019\_7528-model1.21 | -18.6365 |
|  | XP\_065057019\_7528-model1.22 | -18.6347 |
|  | XP\_065057019\_7528-model1.23 | -18.632 |
|  | XP\_065057019\_7528-model1.24 | -18.6308 |
|  | XP\_065057019\_7528-model1.25 | -18.6201 |
|  | XP\_065057019\_7528-model1.26 | -18.6179 |
|  | XP\_065057019\_7528-model1.27 | -18.6153 |
|  | XP\_065057019\_7528-model1.28 | -18.6109 |
|  | XP\_065057019\_7528-model1.29 | -18.5986 |
|  | XP\_065057019\_7528-model1.30 | -18.5929 |
|  | XP\_065057019\_7528-model1.31 | -18.59 |
|  | XP\_065057019\_7528-model1.32 | -18.5792 |
|  | XP\_065057019\_7528-model1.33 | -18.5792 |
|  | XP\_065057019\_7528-model1.34 | -18.5754 |
|  | XP\_065057019\_7528-model1.35 | -18.5754 |
|  | XP\_065057019\_7528-model1.36 | -18.5748 |
|  | XP\_065057019\_7528-model1.37 | -18.5733 |
|  | XP\_065057019\_7528-model1.38 | -18.5724 |
|  | XP\_065057019\_7528-model1.39 | -18.5694 |
|  | XP\_065057019\_7528-model1.40 | -18.569 |
|  | XP\_065057019\_7528-model1.41 | -18.5684 |
|  | XP\_065057019\_7528-model1.42 | -18.5684 |
|  | XP\_065057019\_7528-model1.43 | -18.5684 |
|  | XP\_065057019\_7528-model1.44 | -18.5684 |
|  | XP\_065057019\_7528-model1.45 | -18.5684 |
|  | XP\_065057019\_7528-model1.46 | -18.563 |
|  | XP\_065057019\_7528-model1.47 | -18.5447 |
|  | XP\_065057019\_7528-model1.48 | -18.5419 |
|  | XP\_065057019\_7528-model1.49 | -18.5419 |
|  | XP\_065057019\_7528-model1.50 | -18.5362 |
|  | XP\_065057019\_7528-model1.51 | -18.5353 |
|  | XP\_065057019\_7528-model1.52 | -18.528 |
|  | XP\_065057019\_7528-model1.53 | -18.5267 |
|  | XP\_065057019\_7528-model1.54 | -18.5239 |
|  | XP\_065057019\_7528-model1.55 | -18.5239 |
|  | XP\_065057019\_7528-model1.56 | -18.5197 |
|  | XP\_065057019\_7528-model1.57 | -18.5161 |
|  | XP\_065057019\_7528-model1.58 | -18.5159 |
|  | XP\_065057019\_7528-model1.59 | -18.4977 |
|  | XP\_065057019\_7528-model1.60 | -18.4829 |
|  | XP\_065057019\_7528-model1.61 | -18.4829 |
|  | XP\_065057019\_7528-model1.62 | -18.4829 |
|  | XP\_065057019\_7528-model1.63 | -18.4829 |
|  | XP\_065057019\_7528-model1.64 | -18.4723 |
|  | XP\_065057019\_7528-model1.65 | -18.4656 |
|  | XP\_065057019\_7528-model1.66 | -18.4612 |
|  | XP\_065057019\_7528-model1.67 | -18.4576 |
|  | XP\_065057019\_7528-model1.68 | -18.4576 |
|  | XP\_065057019\_7528-model1.69 | -18.4562 |
|  | XP\_065057019\_7528-model1.70 | -18.4562 |
|  | XP\_065057019\_7528-model1.71 | -18.4528 |
|  | XP\_065057019\_7528-model1.72 | -18.4364 |
|  | XP\_065057019\_7528-model1.73 | -18.4339 |
|  | XP\_065057019\_7528-model1.74 | -18.4276 |
|  | XP\_065057019\_7528-model1.75 | -18.426 |
|  | XP\_065057019\_7528-model1.76 | -18.426 |
|  | XP\_065057019\_7528-model1.77 | -18.4129 |
|  | XP\_065057019\_7528-model1.78 | -18.4075 |
|  | XP\_065057019\_7528-model1.79 | -18.4006 |
|  | XP\_065057019\_7528-model1.80 | -18.3998 |
|  | XP\_065057019\_7528-model1.81 | -18.3998 |
|  | XP\_065057019\_7528-model1.82 | -18.2965 |
| # cluster 2 - Size: 12 |  |  |
|  | XP\_065057019\_7528-model2 | -14.9047 |
|  | XP\_065057019\_7528-model2.1 | -14.7394 |
|  | XP\_065057019\_7528-model2.2 | -14.5802 |
|  | XP\_065057019\_7528-model2.3 | -14.2568 |
|  | XP\_065057019\_7528-model2.4 | -14.1768 |
|  | XP\_065057019\_7528-model2.5 | -14.1495 |
|  | XP\_065057019\_7528-model2.6 | -14.1106 |
|  | XP\_065057019\_7528-model2.7 | -14.0352 |
|  | XP\_065057019\_7528-model2.8 | -13.9709 |
|  | XP\_065057019\_7528-model2.9 | -13.8325 |
|  | XP\_065057019\_7528-model2.10 | -13.6954 |
|  | XP\_065057019\_7528-model2.11 | -13.5938 |
| # cluster 3 - Size: 1 |  |  |
|  | XP\_065057019\_7528-model3 | -13.8344 |
| # cluster 4 - Size: 2 |  |  |
|  | XP\_065057019\_7528-model4 | -13.8178 |
|  | XP\_065057019\_7528-model4.1 | -13.4562 |
| # cluster 5 - Size: 1 |  |  |
|  | XP\_065057019\_7528-model5 | -13.6986 |
| # cluster 6 - Size: 1 |  |  |
|  | XP\_065057019\_7528-model6 | -13.3097 |
|  |  |  |
